# University students’ opinion on gamete donor identification regimes

**DOI:** 10.1007/s10815-023-02832-w

**Published:** 2023-05-26

**Authors:** Ana Nogueira, Omar Ammar, Enes Bilir, Lavinia Iftene, Ignácio Torrero, Nathan Ceschin, Cristina Nogueira-Silva, Pedro Brandão

**Affiliations:** 1 Medical School, University of Minho, Universidade Do Minho, Campus de Gualtar, 4710-057 Braga, Portugal; 2 Ar-Razi Private Hospital, 60 Street, Ramadi, Iraq; 3 School of Medicine, Acibadem Mehmet Ali Aydinlar University, Istanbul, Turkey; 4 Clinical Hospital Nicolae Malaxa, Bucharest, Romania; 5 University Cardenal Herrera, Carrer Lluís Vives, 1, 46115 Alfara del Patriarca, Valencia, Spain; 6 Feliccità Fertility Institute, Rua Conselheiro Dantas, 1154-Prado Velho, Curitiba, Paraná 80220-191 Brazil; 7 Ginemed Porto, Avenida da Boavista, 1243, 4100-130 Porto, Portugal

**Keywords:** Anonymity, Fertility, Oocyte donation, Reproduction, Reproductive techniques, Sperm donation

## Abstract

**Purpose:**

This study aims to evaluate the opinion of university students about the identification or nonidentification of gamete donation and the probability of donation according to the different regimes.

**Methods:**

This was a cross-sectional observational study based on an online anonymous survey including questions about sociodemographic data, reasons for considering donations, information about the donation process and legislation, and their opinions about the different regimes and how they would influence donations.

**Results:**

In total, 1393 valid responses were obtained, with a mean age of 24.0 years (SD = 4.8), most of the respondents being female (68.5%), living in a relationship (56.7%), and without children (88.4%). The main reasons for considering donation would be altruism and monetary compensation. Overall, it was found that participants were poorly informed about the donation procedure and legislation. Students revealed preference for nonidentified donation, and they were less likely to donate in an open identity regime.

**Conclusion:**

Most university students consider themselves poorly informed about gamete donation, express a preference for nonidentified gamete donation, and would less likely donate on an open identity basis. Thus, an identified regime may be less attractive to potential donors and lead to a decrease in the availability of gamete donors.

## Introduction

Gamete donation is a commonly used procedure to help individuals or couples achieve parenthood when their own gametes are not available or viable [1]. This includes those with fertility issues, risk of genetic conditions, single people, same-sex couples, and transgender individuals or couples [2].

The number of children born using donated gametes has been on the rise, possibly due to greater social acceptance of the so called “new family types” such as single parents or same-sex couples, which may explain this [3–6].

Gamete donation is a particularly sensitive issue as there is no genetic link between the offspring and one or both parents [7]. Thus, it brings complex ethical issues, such as the anonymity of the donation.

Anonymity prevents the donor’s identity, but in some countries, anonymity can be broken in certain medical conditions. Anonymity prevents the donor’s identity from being revealed, both to the recipients and the offspring. Likewise, the donor cannot choose the recipient and recipients do not choose their donor. Nevertheless, in most countries, anonymity may exceptionally be broken in the presence of certain medical conditions [8]. In 2022, the ASRM published an update on the terminology to be used concerning the matter. Donors who were previously described as known may be referred to as directed (identified) donors, and those previously described as anonymous may be referred to as nonidentified donors [9].

The debate surrounding the nonidentification of donation is primarily centered on the right to privacy and autonomy of both parents and donors, as well as the right of the children to know their origins. The latter is one of the main arguments against the nonidentification of donation [2, 10]. Given that the offspring is the only party involved who does not have a word when it comes to deciding the regime of donation, protecting their best interest is of utmost importance [11–13]. Skeptics of the nonidentification regime claim that it calls into question fundamental interests of the offspring concerning family relationships, their identity, and health [11, 14]. Knowing one’s heritage can be important for reproductive decisions, diagnosis, and preventive strategies [15].

Recipients tend to choose a nonidentified donor to avoid involving a third party in their family [16–19]. Many parents, especially heterosexual couples, find it difficult to disclose the use of donated gametes to their children due to fear of disrupting family ties or lack of knowledge on how and when to address the issue. Consequently, many people remain unaware of the nature of their conception [20–23].

Studies conducted in countries with a nonidentification regime reported that a large proportion of donors prefer nonidentified donation and they would not donate if nonidentification were to end. An open identity regime may lead to greater difficulty in recruiting donors, increasing the gap between demand and supply [24–26]. Contrarily, some donors feel a responsibility towards the resulting offspring, agreeing to the disclosure of their identity out of a desire for future contact with the offspring. In these cases, a waiting period is established before donors are identified in order to protect the privacy of the family and allow the establishment of stable relationships between the children and their parents [27, 28].

Legislation regarding gamete donation has changed over the years. The first country to suspend nonidentification was Sweden in 1984. Since then, several countries have shifted towards a direct (identified) regime [29, 30].

Currently, there are different regimes for gamete donation in force worldwide. For example, in the Netherlands, donations occur in a full identification regime; in Sweden, the UK, or Portugal, donations occur in a partially identified regime (identified for recipients but not for the offspring when they reach adulthood); in Spain, nonidentification is mandatory; in Denmark, Belgium, or Germany, donors can choose whether to be identified or not [3, 30, 31].

With the increasing number of people opting for direct-to-consumer genetic testing and the advancements in genetic analysis leading to greater accuracy, the risk of identification has grown exponentially. Therefore, it is reasonable to assume that nowadays complete nonidentification for the future cannot be guaranteed [8, 32].

It is important to understand the opinion of potential donors about different regimes of donation and how they may affect gamete donation. Since gamete donors, in particular oocyte donors, are young and healthy people, usually in their 20 s and early 30 s, university students are a good sample representing potential donors.

This study aims to assess the opinion of university students about the regimes of gamete donation.

## Methods

This is an observational cross-sectional study based on surveys, distributed to university students from June to December 2021.

The criteria for inclusion were university students, with Portuguese nationality or residency in Portugal, aged between 18 and 35 years old.

The study protocol was approved by the local Ethics Committee. The participants’ anonymity and the confidentiality of the data were guaranteed at all stages of the study.

The survey was composed of 26 questions, available as a Google Forms®. At the beginning of the survey, there was a brief explanation of the purpose of the study and the concepts of donor, recipient, and offspring were defined. Respondents were reassured that their participation was voluntary and anonymous. Participants were asked to expressly give their informed consent.

The initial questions focused on sociodemographic data: age, nationality, country of residence, gender, relationship status, level of studies, the field of study, and existence of children.

The survey aimed to gather insights on several aspects related to gamete donation, including the motivations for considering gamete donation, knowledge of the donation procedure and relevant legislation, the impact of different identification regimes on donation, and attitudes towards identity disclosure among the three parties involved (donors, recipients, and offspring). Additionally, we analyzed differences in responses based on gender, relationship status, level of education, and parenthood. Finally, we sought to understand the participants’ opinions on the possibility of contact with someone born from their donated gametes in the future.

The sampling process was based on the snowball method. Data were collected through the online survey shared on social networks and sent by email to students from different Portuguese universities. Participants were asked to share the survey with their peers.

The answers were stored in an IBM SPSS® database. Most answers were on a Likert scale, so most of the outcomes were assessed by describing absolute and relative numbers (percentages). Comparisons between the different groups (categorical variables—gender, current relationship, level of studies, and own children) were performed using the chi-square test.

## Results

In total, 1577 answers were obtained, of which 1393 were considered valid after applying the criteria of inclusion.

Table 1 shows the participants’ sociodemographic data and Table 2 the current level and field of study of the participants.

**Table 1:** Descriptive analysis of the sociodemographic data of the sample

Variable		Frequency (*n*)	Percentage (%)
Number of participants		1393	
Age	18 to 25	851	61.1%
	26 to 35	542	38.9%
Gender	Female	954	68.5%
	Male	439	31.5%
Current relationship	No partner	631	45.3%
	In a relationship (not married)	599	43.0%
	Married	163	11.7%
Children	No	1231	88.4%
	Yes	162	11.6%
Nationality	Portugal	1351	97.0%
	Brazil	30	2.2%
	Others	12	0.8%
Residence	Portugal	1361	97.8%
	United Kingdom	16	1.1%
	Others	16	1.1%
Level of current studies	Graduation	724	52.0%
	Postgraduate courses	95	6.8%
	Master degree	500	35.9%
	PhD	72	5.2%
	Post Doc	2	0.1%
Field of study	Health Sciences	476	34.2%
	Engineering	180	12.9%
	Psychology, Sociology, or Philosophy	113	8.1%
	Economics and Management	111	8.0%
	Basic Sciences	71	5.1%
	Education	64	4.6%
	Law	49	3.5%
	Others	329	23.6%
Previous donation	No	1382	99.2%
	Yes	11	0.8%
Regime of previous donation	Offspring may know the identity of the donor	8	72.7%
	Fully nonidentified	3	27.3%
	Recipients may know the identity of the donor	1	9.1%
	Recipients may choose the donor	1	9.1%

**Table 2:** Possible future donation

Variable		Frequency (*n*)	Percentage (%)
Number of participants		1393	
Consider future donation	No	985	70.7%
	Yes	408	29.3%
Reasons to consider donation	Help heterosexual couples	344	82.9%
	Help same-sex couples	231	55.7%
	Help avoiding transmission of genetic disorder couples	209	50.4%
	Monetary reward	193	46.5%
	Help single people	189	45.5%
	Other	7	1.7%

Most of the participants were aged between 18 and 25 years (61.1%), 68.5%, 45.3% were in no relationship, and only 11.6% had children. More than half were undergraduate students (52.0%), and the most frequent fields of study were Health Sciences (34.2%), Engineering (12.9%), Psychology, Sociology or Philosophy (8.1%), and Basic Sciences (5.1%). Only 0.8% of the participants had donated gametes before, 27.3% of them in a fully anonymous regime.

Regarding their motivations for a potential donation, 82.9% would donate to help heterosexual couples overcome infertility, 55.7% to help same-sex couples to have children, 50.4% to help avoid the transmission of genetic disorders, 46.5% for monetary compensation, and 45.5% to help single people to have children (Table 2).

Students were poorly informed regarding current legislation on gamete donation, 71.1% of the students said they were not informed at all. Regarding the nonidentification of donation in Portugal, 65.8% of the participants do not know whether the procedure was identified or not and only 5.9% thought it was identified. Regarding the gamete donation procedure itself, 71.1% of the students stated that they were not informed at all (Table 3).

**Table 3:** Level of knowledge about legislation, national anonymity regime, and the procedure of gamete donation

Variable		Frequency (*n*)	Percentage (%)
Number of participants		1393	
Knowledge about national legislation	Not informed	990	71.1%
	Moderately informed	394	28.3%
	Very well informed	9	0.6%
Knowledge about the procedure	Not informed	991	71.1%
	Moderately informed	388	27.9%
	Very well informed	14	1.0%

**Fig. 1:**
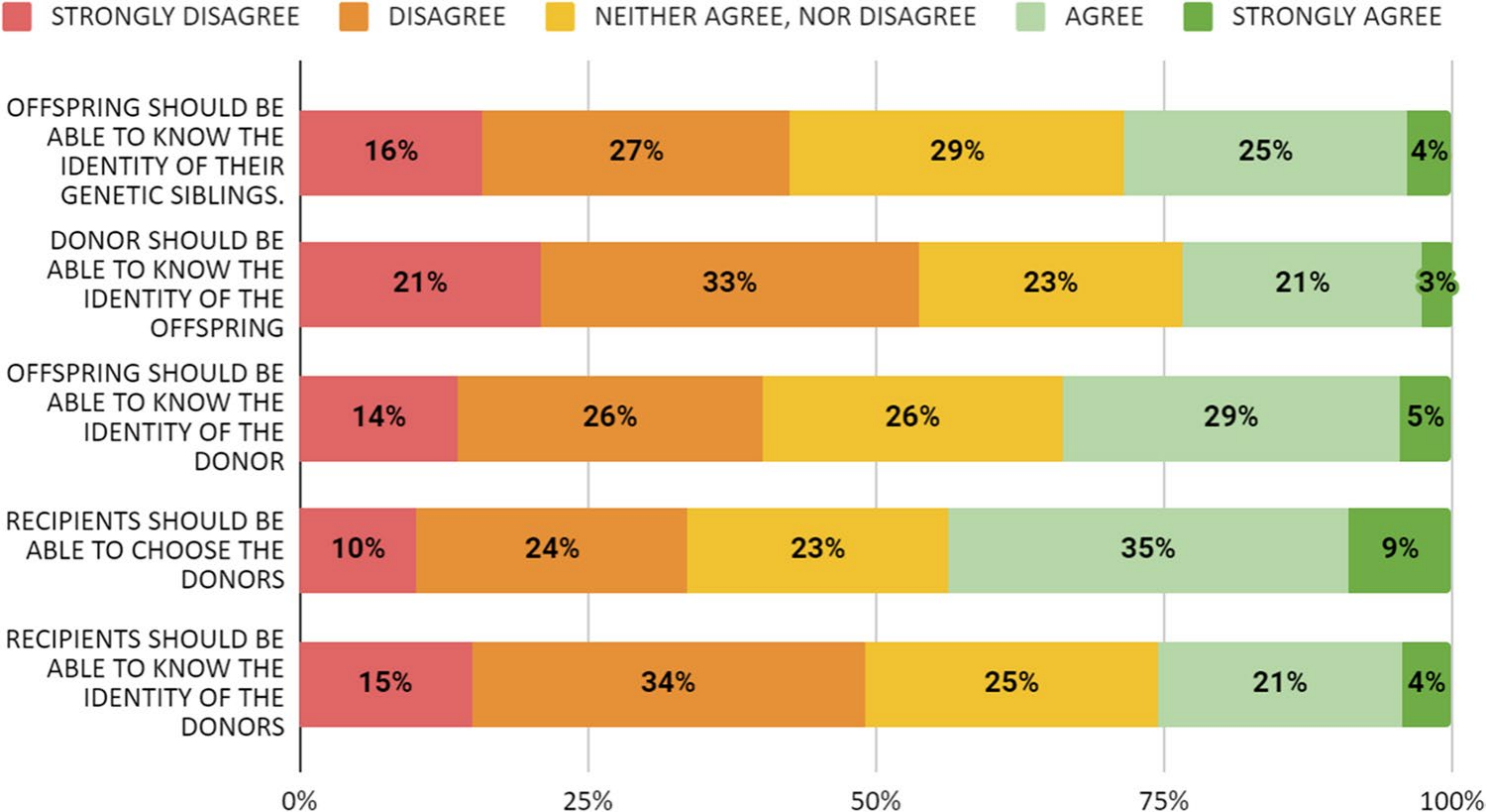
Participants’ opinion regarding the disclosure of the identity of the three parties (donor, recipients, and offspring) between each other

Figure 1 shows the opinion of the participants concerning the different regimes of donation.

When asked if recipients should have the right to know the identity of the donor, 49.2% of participants disagreed or strongly disagreed, 25.4% expressed a neutral opinion, and the remaining 25.4% expressed a favorable opinion (Fig. 1). There was a higher percentage of disagreement among students with a partner (53.5%; *p* < 0.001) and postgraduate education (57%; *p* < 0.001), but no differences were observed between genders or participants with or without children.

Likewise, if recipients should have the right to choose the donor, 43.7% of the participants had a favorable opinion, 22.6% were neutral, and 33.7% were against it. Only the participants with a partner showed more disagreement concerning this aspect (37%, *p* = 0.002), with no differences according to the level of studies, sex, or previous children (Fig. 1).

About the offspring, 40.1% were against the offspring having the right to know the identity of the donor and 33.7% were in favor (Fig. 1). On this topic, participants with a partner (44%; *p* = 0.001), postgraduation (46.8%; < 0.001), and children (51.9%; *p* = 0.005) showed more disagreement, with no differences regarding gender.

In addition, 42.6% of the participants expressed an opinion against the right of the offspring to know their genetic siblings and 28.4% were of a favorable opinion (Fig. 1). On this matter, opposition was significantly higher in participants with a partner (46.6%, *p* < 0.001), of postgraduate education (48.9%; *p* < 0.001), and with children (53.1%; *p* = 0.017).

Concerning the donors’ right to know the identity of the offspring, 53.7% of the participants were against while 23.4% agreed or strongly agreed (Fig. 1). Again, only participants with a partner (57.9%; *p* < 0.001) and postgraduate (60.4%; *p* < 0.001) showed greater disagreement.

**Fig. 2:**
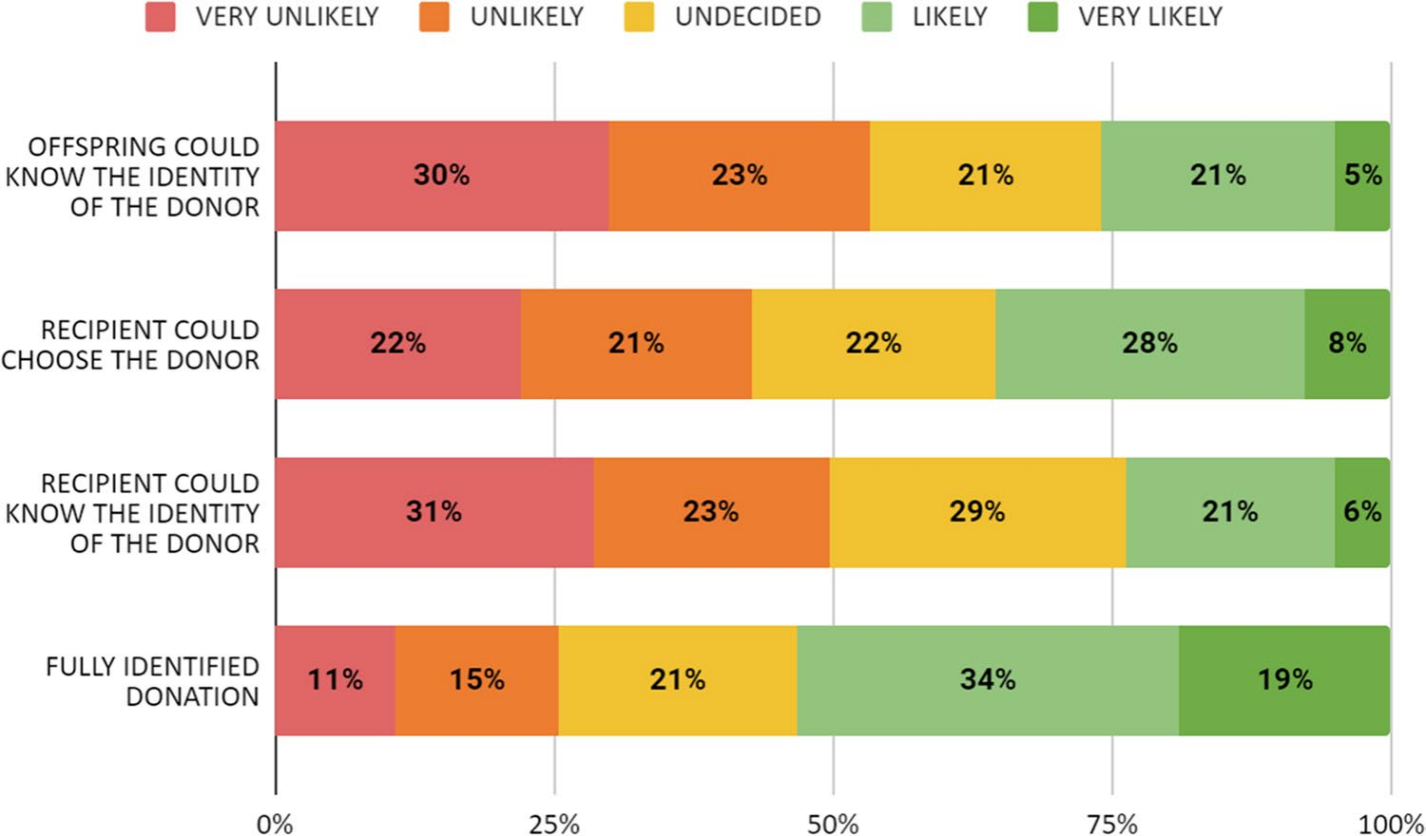
Likelihood of donation under different anonymity regimes

In a total nonidentifed regime, most participants (53.2%) considered themselves as likely or very likely to donate gametes, while 25.4% said it is unlikely or very unlikely to donate gametes (Fig. 2). The undergraduate students were slightly more willing to donate under this regime (55.1% would probably or very probably donate, *p* = 0.048), but no differences were found regarding gender, previous children, or current relationship status.

In case recipients had the right to know the identity of the donor, 54.6% of the participants said it would be unlikely or very unlikely to donate gametes, while 26.1% would likely or very likely donate (Fig. 2). In this case, a higher percentage of unpartnered (30.3%, *p* = 0.005) and undergraduate education (29.1%, *p* < 0.001) participants answered it was likely or very likely to donate. No differences were found concerning sex or relationship status.

Likewise, in the event recipients could choose the donors, 42.6% stated that they would unlikely or very unlikely donate, while 35.3% stated that donation was likely or very likely (Fig. 2). There were no differences regarding gender, level of studies, current relationship status, or previous children.

If the offspring could know the identity of the donor, 53.4% of the participants were unlikely or very unlikely to donate gametes while 25.9% were likely or very likely (Fig. 2). Only undergraduate students responded that they would likely donate (29.8%, *p* < 0.001).

Of those who would probably/very probably donate if gamete donation was completely nonidentified, 48.2% would not donate if the offspring had the right to know the identity of the donor. On the other hand, of those who would probably/very probably donate if the offspring had the right to know the identity of the donor, 15.8% would not donate if the gamete donation was completely nonidentified. Interestingly, however, of those who agree that the offspring should have the right to know the identity of the donor, only 5.7% would donate under those conditions, but 59.7% of them would donate if the donation was completely nonidentified.

**Fig. 3:**
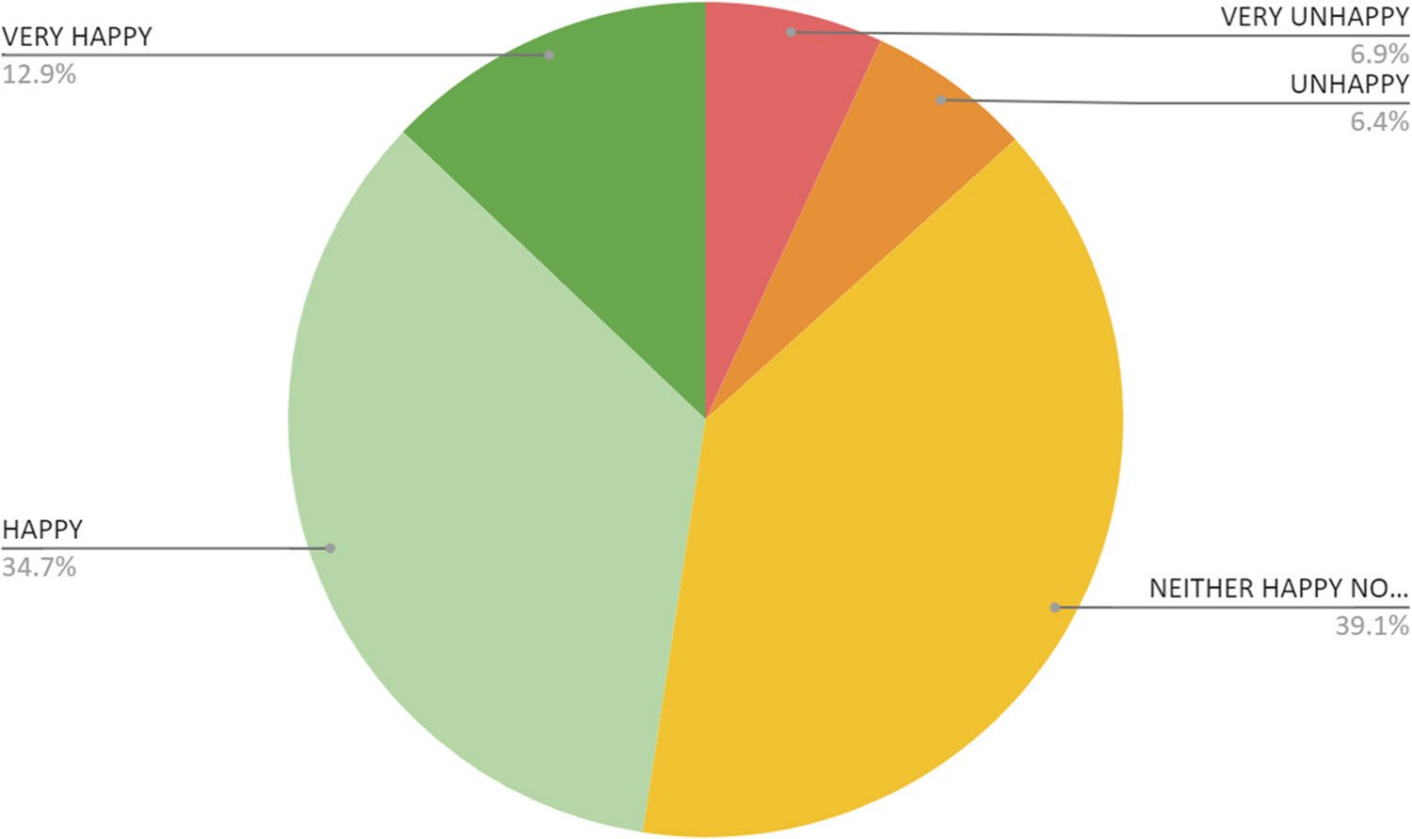
Answer to the question “how happy would you be if the offspring contacted you?”

Concerning a possible future contact with the offspring, 47.6% of the participants stated that they would be happy or very happy with that, while 13.3% would be unhappy or very unhappy (Fig. 3). A higher percentage of undergraduates assume that they would be happy or very happy in this situation (51.9%, *p* = 0.003), but there were no differences according to gender, relationship status, or previous children.

## Discussion

The incidence of infertility has progressively grown over the years. Likewise, same-sex couples and single women who cannot procreate without the use of assisted reproduction techniques and donation are of utmost importance [33, 34].

Gamete donation is not exempt from ethical issues, particularly in relation to donor anonymity. The right of the offspring to know their genetic origin is one of the main arguments against anonymity and the main reason for the adoption of open identity policies [2, 10]. There is some fear that this new paradigm may affect the number of donors; thus, the importance of understanding it may influence the decision to donate.

University students are an important group of potential donors. In this study, 1393 students were included, of which only 11 reported a previous gamete donation (0.8%). On the other hand, 29.3% of participants report having already considered gamete donation at some point. Thus, it is necessary to understand which measures should be adopted to recruit potential donations.

Similar to previously published data, the most mentioned reasons were altruism (helping couples overcome infertility, helping same-sex couples have children, helping people at high risk of genetic diseases if helping single man/women to have children) and monetary compensation. Interestingly, none of the participants expressed the desire to check their fertility as a motivation for donation [26, 35, 36]. A significant difference was observed in the percentage of respondents who would consider helping opposite-sex couples overcome infertility compared to those who would assist same-sex couples or single patients. This may reflect a lack of information and awareness among the population regarding the possibility of using assisted reproductive techniques by same-sex couples or single individuals. This raises concerns about the need to educate the public about the positive parenting potential of non-heteronormative families [37, 38].

The low number of donations observed in this study may be partially explained by the lack of information on this issue. The results show that most of the students were not informed about current legislation and the procedure for gamete donation. The promotion of informative actions, counseling services, and donor experience-sharing sessions could be useful to increase the number of donors [39].

The literature on the general population’s opinion regarding gamete donation and more specifically the rights of each party involved is scarce. We found that 49.2% of the participants were against the right of recipients to know the identity of the donor, 40.1% were against the right of the offspring to know the identity of the donor, 53.7% were against the right of donors to know the identity of the offspring, and 42.6% were against the right of the offspring to know the genetic siblings. On the other hand, 43.7% of the participants advocated that recipients should have the right to choose the donor. Interestingly, students with a partner, with higher qualifications, or with children tended to be more opposed to identified regimes.

Previous studies have suggested that a significant proportion of gamete donors prefer to remain nonidentified and may be less likely to donate if identification were mandated. This could result in challenges with donor recruitment and increased demand for gametes [24–26, 40]. In parallel, a study conducted in Belgium on male higher education students revealed that 34.3% of participants have ever considered a donation, and of these, 61.7% would not reveal information about themselves, and only 1 in 4 would consider a donation if their identity could be revealed [41].

Overall, participants express a clear preference for nonidentified regimes. In a regime of complete nonidentification of the donation, 53.2% of the participants reported as likely/very likely to donate, while a much lower percentage would consider a donation in an open identity regime, especially if the recipients could choose donors. Postgraduate students and students with a partner were even less likely to donate in an identified regime.

Furthermore, 48.2% of the participants who would donate under a fully nonidentified regime would not donate if the offspring could know the identity of the donor. In addition, most of the participants who believe the offspring should have the right to know the identity of the donor would not donate in an open identity regime. These findings may suggest that identified regimes may lead to fewer available donors.

Interestingly, despite the overall preference for nonidentified regimes, 47.6% of the participants mentioned they would be happy or very happy with future contact with the offspring resulting from their donated gametes.

One of the key messages from this study is that the general population is not sufficiently aware of gamete donation, particularly what matters about donors’ identification. In our sample, the majority of respondents would rather donate anonymously. In today’s world, where information flows freely and genetic databases are prevalent worldwide, the promises of anonymity provided by current gamete banks or assisted reproduction facilities, which use them as a means to attract donors, must be carefully considered. These results suggest that the donor recruitment policies should be carefully rethought and education and guidance programs should be implemented, highlighting the potential scenario of disclosure [8, 42].

This study was conducted on a large sample size, with 1393 valid responses, which is a representative number of university students. The sample included was diverse considering various demographic factors, such as age, gender, relationship status, level of studies, and the existence of children, which can provide a broad understanding of the opinions of the participants. Nevertheless, it is a cross-sectional and observational study, based on self-reported data, which may be subject to inaccuracies in participants’ responses. The sampling process was based on the snowball method, which on the one hand led to a large sample size but on the other hand may lead to sampling bias, as participants may have shared the survey with peers who have similar opinions or characteristics. Finally, the study focused on university students in Portugal, limiting its generalizability to other populations or cultures. Most students did not show interest in donating, which may inaccurately reflect the reality of the opinion of potential donors.

## Conclusion

University students have shown a preference for nonidentified gamete donation regimes. The main reasons they consider gamete donation are altruism and monetary compensation. However, most students consider themselves poorly informed about the legislation and process of gamete donation.

Although opinions vary, the majority of students disagree with disclosing identities in gamete donation and are less likely to donate on an open identity basis. This suggests that an identified regime may be less attractive to potential donors, which could lead to a decrease in the availability of gamete donors over time.

Moreover, considering the limited awareness of donation policies and the increasing availability of direct-to-consumer databases that compromise anonymity, it is crucial to carefully reconsider donor recruitment policies and establish education and guidance programs that emphasize the potential risks of disclosure.

## Data Availability

All data were anonymously retrieved. The personal data was treated according to Regulation EU 2016/679 of the European Parliament and of the Council of April 27, 2016, concerning the protection of natural persons with regard to the processing of personal data and the free circulation of such data.

## References

[1] Maung HH (2019). Ethical problems with ethnic matching in gamete donation. J Med Ethics.

[2] Melo-Martín ID (2014). The ethics of anonymous gamete donation: is there a right to know one’s genetic origins?. Hastings Cent Rep.

[3] Brandão P (2022). European policies on same-sex relationships, adoption and assisted reproduction. Int J Reprod Contracept Obstet Gynecol.

[4] Golombok S (2017). Parenting in new family forms. Curr Opin Psychol Elsevier Ltd.

[5] Greenfeld DA (2005). Reproduction in same sex couples: quality of parenting and child development. Curr Opin Obstet Gynecol.

[6] Imrie S, Jadva V, Fishel S, Golombok S (2019). Families created by egg donation: parent-child relationship quality in infancy. Child Dev.

[7] ESHRE Task Force on Ethics and Law (2002). III Gamete and embryo donation. Hum Reprod..

[8] Pennings G (2019). Genetic databases and the future of donor anonymity. Hum Reprod Oxf Engl.

[9] Updated terminology for gamete and embryo donors (2022). directed (identified) to replace “known” and nonidentified to replace “anonymous”: a committee opinion. Fertil Steril.

[10] Nelson MK, Hertz R, Kramer W (2016). Gamete donor anonymity and limits on numbers of offspring: the views of three stakeholders. J Law Biosci.

[11] de Melo-Martín I (2016). How best to protect the vital interests of donor-conceived individuals: prohibiting or mandating anonymity in gamete donations?. Reprod Biomed Soc Online.

[12] Macpherson I (2019). Ethical reflections about the anonymity in gamete donation. Hum Reprod.

[13] Brandão P, Garrido N (2022). Commercial surrogacy: an overview. Rev Bras Ginecol E Obstetrícia RBGO Gynecol Obstet.

[14] Ravitsky V (2012). Conceived and Deceived: The Medical Interests of Donor-Conceived Individuals. Hastings Cent Rep.

[15] Ravitsky V (2017). The right to know one’s genetic origins and cross-border medically assisted reproduction. Isr J Health Policy Res.

[16] Bracewell-Milnes T, Saso S, Bora S, Ismail AM, Al-Memar M, Hamed AH (2016). Investigating psychosocial attitudes, motivations and experiences of oocyte donors, recipients and egg sharers: a systematic review. Hum Reprod Update.

[17] Ezugwu EC, Eleje GU, Iyoke CA, Mba SG, Nnaji HC, Enechukwu CI (2018). Preference for anonymity in sperm donation for artificial insemination: an experience from low-resource settings in Nigeria. Patient Prefer Adherence.

[18] Laruelle C, Place I, Demeestere I, Englert Y, Delbaere A (2011). Anonymity and secrecy options of recipient couples and donors, and ethnic origin influence in three types of oocyte donation. Hum Reprod.

[19] da Silva SP, Freitas CD, Baía I, Samorinha C, Machado H, Silva S (2019). Gamete donation: (un)answered social and ethical issues in portugal. Cad Saude Publica.

[20] de Melo-Martín I, Rubin LR, Cholst IN (2018). “I want us to be a normal family”: toward an understanding of the functions of anonymity among U.S. oocyte donors and recipients. AJOB Empir Bioeth..

[21] Lampic C, Skoog Svanberg A, Sorjonen K, Sydsjö G (2021). Understanding parents’ intention to disclose the donor conception to their child by application of the theory of planned behaviour. Hum Reprod.

[22] Tallandini MA, Zanchettin L, Gronchi G, Morsan V (2016). Parental disclosure of assisted reproductive technology (ART) conception to their children: a systematic and meta-analytic review. Hum Reprod.

[23] Brandão P, Ceschin N, Sandvik B, Paolelli S, Doblinger J, Reis-Soares S, et al. Female couples undergoing assisted reproduction - choices and the importance of pregnancy and genetic. JBRA Assist Reprod. 2023 (in press). 10.5935/1518-0557.20230007 PMC10712815 37257076

[24] Cordier C, Ducrocq B, Fry J, Catteau-Jonard S (2020). Views of French oocyte donors at least 3 years after donation. Reprod Biomed Online.

[25] Mahieu F, Decleer W, Osmanagaoglu K, Provoost V (2019). Anonymous sperm donors’ attitude towards donation and the release of identifying information. J Assist Reprod Genet.

[26] Thijssen A, Provoost V, Vandormael E, Dhont N, Pennings G, Ombelet W (2017). Motivations and attitudes of candidate sperm donors in Belgium. Fertil Steril.

[27] Kirkman M, Bourne K, Fisher J, Johnson L, Hammarberg K (2014). Gamete donors’ expectations and experiences of contact with their donor offspring. Hum Reprod.

[28] Lampic C, Svanberg AS, Sydsjö G (2014). Attitudes towards disclosure and relationship to donor offspring among a national cohort of identity-release oocyte and sperm donors. Hum Reprod.

[29] Bujan L, Lannou DL, Kunstmann JM (2012). Anonymat du don de gamètes. Gynecol Obstet Fertil Elsevier.

[30] Calhaz-Jorge C, Geyter ChD, Kupka MS, Wyns C, Mocanu E, Motrenko T (2020). Survey on ART and IUI: legislation, regulation, funding and registries in European countries. Hum Reprod Open..

[31] Wyns C, De Geyter C, Calhaz-Jorge C, Kupka MS, Motrenko T, Smeenk J (2021). ART in Europe, 2017: results generated from European registries by ESHRE. Hum Reprod Open.

[32] Harper JC, Kennett D, Reisel D (2016). The end of donor anonymity: how genetic testing is likely to drive anonymous gamete donation out of business. Hum Reprod.

[33] Areias J, Gato J, Moura-Ramos M (2022). Motivations and attitudes of men towards sperm donation: whom to donate and why?. Sex Res Soc Policy.

[34] Brandão P, Ceschin N, Gómez VH (2022). The pathway of female couples in a fertility clinic. Rev Bras Ginecol E Obstet.

[35] Hedrih A, Hedrih V (2012). Attitudes and motives of potential sperm donors in Serbia. Vojnosanit Pregl.

[36] Pennings G, de Mouzon J, Shenfield F, Ferraretti AP, Mardesic T, Ruiz A (2014). Socio-demographic and fertility-related characteristics and motivations of oocyte donors in eleven European countries. Hum Reprod.

[37] Brandão P, de Pinho A, Ceschin N, Sousa-Santos R, Reis-Soares S, Bellver J (2022). ROPA – lesbian shared in vitro fertilization – ethical aspects. Eur J Obstet Gynecol Reprod Biol.

[38] Pennings G (2011). Evaluating the welfare of the child in same-sex families. Hum Reprod Oxf Engl.

[39] Carolino N, Galhardo A, Cunha M (2019). Atitudes face à doação de gâmetas e gestação de substituição. Rev Port Investig Comportamental E Soc.

[40] Bay B, Larsen PB, Kesmodel US, Ingerslev HJ (2014). Danish sperm donors across three decades: motivations and attitudes. Fertil Steril.

[41] Provoost V, Van Rompuy F, Pennings G (2018). Non-donors’ attitudes towards sperm donation and their willingness to donate. J Assist Reprod Genet.

[42] Neyroud A-S, Roche M, Domin M, Jaillard S, Ravel C (2020). L’anonymat du don de gamètes à l’heure des tests génétiques. Gynécol Obstét Fertil Sénologie.

